# The Tri-ponderal Mass Index is associated with adiposity in adolescent type 2 diabetes mellitus: a cross-sectional analysis

**DOI:** 10.1038/s41598-021-88705-7

**Published:** 2021-04-27

**Authors:** Haifa Alfaraidi, Brandy Wicklow, Allison B. Dart, Elizabeth Sellers, Jonathan McGavock, Lehana Thabane, M. Constantine Samaan

**Affiliations:** 1 Department of Pediatrics, McMaster University, 1280 Main Street West, HSC-3A57, Hamilton, ON L8S 4K1 Canada; 2 Division of Pediatric Endocrinology, McMaster Children’s Hospital, Hamilton, Ontario Canada; 3 Department of Pediatrics and Child Health, University of Manitoba, Winnipeg, Manitoba Canada; 4 Children’s Hospital Research Institute of Manitoba, University of Manitoba, Winnipeg, Manitoba Canada; 5 Department of Health Research Methods, Evidence and Impact, McMaster University, Hamilton, Ontario Canada; 6 Department of Anesthesia, McMaster University, Hamilton, Ontario Canada; 7 Centre for Evaluation of Medicines, Hamilton, Ontario Canada; 8 Biostatistics Unit, St. Joseph’s Healthcare, Hamilton, Ontario Canada; 9 Michael G. DeGroote School of Medicine, McMaster University, Hamilton, Ontario Canada

**Keywords:** Endocrinology, Endocrine system and metabolic diseases, Diseases, Endocrine system and metabolic diseases

## Abstract

Pediatric type 2 diabetes mellitus (T2DM) patients are often overweight or obese, yet there are no validated clinical measures of adiposity to stratify cardiometabolic risk in this population. The tri-ponderal mass index (TMI, kg/m^3^) has recently been reported as a measure of adiposity in children, but there has been no validation of the association of TMI with adiposity in pediatric T2DM. We hypothesized that in children with T2DM, the TMI can serve as a more accurate measure of adiposity when compared to BMI z-score, and that it is associated with components of the metabolic syndrome. This is a cross-sectional secondary data analysis from the Improving Renal Complications in Adolescents with Type 2 Diabetes Through REsearch (iCARE) study (n = 116, age 10.20–17.90 years). Spearman’s correlations and multivariable regression were used in the analyses. When compared to DXA, TMI demonstrated significant correlation with total adiposity versus BMI z-score (TMI r = 0.74, p-value < 0.0001; BMI z-score r = − 0.08, p-value 0.403). In regression analyses, TMI was associated with WHtR (B = 35.54, 95% CI 28.81, 42.27, p-value < 0.0001), MAP dipping (B = 1.73, 95% CI 0.12, 3.33, p-value = 0.035), and HDL (B = − 5.83, 95% CI − 10.13, − 1.54, p-value = 0.008). In conclusion, TMI is associated with adiposity and components of the metabolic syndrome in pediatric T2DM patients.

## Introduction

Pediatric type 2 diabetes mellitus (T2DM) rates are rising around the world, and its emergence has mirrored the global rise in childhood obesity^1–3^. Certain pediatric populations are impacted disproportionately by T2DM rates; for example, in Canada, Indigenous youth account for almost half of all newly diagnosed T2DM cases annually^4^.

Pediatric T2DM is a more aggressive disease than adult T2DM, and may present with several concomitant comorbidities including dyslipidemia, hypertension, non-alcoholic fatty liver disease, and with complications such as early nephropathy^5–8^. Importantly, these patients may have increased carotid intima-media thickness, an early risk marker of future cardiovascular disease^9–11^.

In adult population-based studies, adipose tissue expansion in obesity is an important risk factor in the development of diabetes-related cardiovascular disease via several mechanisms including atherosclerosis, inflammation, and insulin resistance^12–15^. In particular, the expansion of the visceral adipose compartment has been proposed as a stronger predictor of adverse cardiometabolic outcomes when compared to body mass index (BMI) and total adiposity^15–18^. While both waist-to-hip ratio (WHR) and waist-to-height ratio (WHtR) are used as clinical measures of central adiposity, the latter has emerged as a more robust predictor of adiposity and cardiometabolic risk factors in children when compared to BMI^19,20^.

There is a limited set of measures to quantify adiposity in children. The body mass index (BMI) z-score, a clinical measure of obesity in children, often misclassifies children as obese^21^. It is also a poor predictor of change in adiposity over time^22^. Furthermore, the use of dual-energy x-ray absorptiometry (DXA) scans, which is considered the gold standard for measuring adiposity^23,24^ or bioelectrical impedance scales is limited by their cost, unavailability in routine clinical settings, and the specific training and time needed for proper use^25,26^.

Recently, the tri-ponderal mass index (TMI, kg/m^3^) has been proposed as an accurate measure of adiposity in children^27–29^. In healthy children, specific cut-offs have been proposed to diagnose overweight and obesity in one study^27^. There are no data to validate TMI as a measure of adiposity in pediatric T2DM.

We hypothesized that in children with T2DM, TMI has a stronger association with DXA-based adiposity measures than BMI z-score. We also hypothesized that TMI is associated with components of the metabolic syndrome including central adiposity, hypertension, and dyslipidemia.

## Results

### Baseline characteristics

The descriptive characteristics of study participants (n = 116) are reported in Table 1. The majority of participants were female (n = 80, 69.00%). The mean age at study enrollment was 14.60 ± 2.00 years, and the mean age at T2DM diagnosis was 11.90 ± 2.00 years. The majority of participants were from First Nations Community (n = 101, 87.10%); the remainder where of Metis, Southeast Asian, African Canadian and Central American descent.

**Table 1:** Participants’ descriptive characteristics.

Variable	Total (mean, SD)	Female (mean, SD)	Male (mean, SD)
Age at enrollment (years)	14.60 (2.00) (n = 116)	14.50 (2.00) (n = 80)	14.90 (2.10) (n = 36)
Age at diagnosis (years)	11.90 (2.00) (n = 102)	11.80 (2.20) (n = 71)	12.00 (1.60) (n = 31)
Height (cm)	164.60 (9.50) (n = 116)	161.80 (8.00) (n = 80)	170.80 (9.60) (n = 36)
Weight (kg)	83.80 (21.10) (n = 116)	80.80 (20.10) (n = 80)	90.50 (21.80) (n = 36)
BMI z-score	2.53 (1.10) (n = 111)	2.30 (1.00) (n = 76)	2.95 (1.10) (n = 35)
BMI percentile	93.70 (9.20) (n = 116)	94.00 (8.70) (n = 80)	93.00 (10.40) (n = 36)
TMI (kg/m^3^)	18.70 (3.70) (n = 116)	18.90 (3.50) (n = 80)	18.10 (4.00) (n = 36)
WHR	0.99 (0.07) (n = 115)	0.99 (0.07) (n = 79)	1.00 (0.06) (n = 36)
WHtR	0.63 (0.08) (n = 115)	0.64 (0.08) (n = 79)	0.62 (0.09) (n = 36)
Total fat mass (g)	31,118.30 (11,002.00) (n = 116)	31,296.20 (10,354.30) (n = 80)	30,723.10 (12,470.00) (n = 36)
Total lean mass (g)	50,429.00 (11,791.20) (n = 116)	47,165.70 (10,105.70) (n = 80)	57,680.70 (12,160.30) (n = 36)
Total lean + BMC mass (g)	52,326.40 (12,144.40) (n = 116)	48,968.90 (10,408.20) (n = 80)	59,787.60 (12,532.20) (n = 36)
FM%	36.90 (6.10) (n = 115)	38.30 (4.40) (n = 80)	33.50 (8.00) (n = 35)
Heart rate (bpm)	83.00 (9.00) (n = 113)	84.00 (10.00) (n = 78)	81.00 (8.00) (n = 35)
Systolic BP (mmHg)	123.00 (10.00) (n = 113)	121.00 (9.00) (n = 78)	128.00 (11.00) (n = 35)
Diastolic BP (mmHg)	71.00 (6.00) (n = 113)	70.00 (6.00) (n = 78)	72.00 (6.00) (n = 35)
MAP (mmHg)	87.70 (6.40) (n = 113)	86.90 (6.10) (n = 78)	89.50 (6.70) (n = 35)
MAP dipping	11.60 (6.70) (n = 107)	10.80 (6.90) (n = 73)	13.40 (6.20) (n = 34)

*BMI* body mass index, *TMI* tri-ponderal mass index, *WHR* waist-to-hip ratio, *WHtR* waist-to-height ratio, *BMC* bone mineral content, *FM%* fat mass percentage, *bpm* beats per minute, *BP* blood pressure, *mmHg* millimeters of mercury, *MAP* mean arterial pressure, *SD* standard deviation.

Only 14 (12.10%, female = 9 (64.20%)) participants had a normal BMI z-score < 85th percentile, with

28 (24.10%, n = 24 female (85.70%)) having a BMI z-score in the overweight range (85th–< 95th percentile) and 74 (63.80%, n = 47 female (63.50%)) participants with a BMI z-score in the obese range (≥95th percentile).

On assessment of the fat mass, participants had excess total adiposity based on fat mass percentage (FM%) measures on DXA scans (FM% mean 36.90 ± 6.10). In addition, participants had central adiposity with a WHR of 0.99 ± 0.07 and WHtR of 0.63 ± 0.08.

The TMI was 18.70 ± 3.70 kg/m^3^, with female participants having a TMI of 18.90 ± 3.50 kg/m^3^. The 85th percentile for TMI for the female participants in our study was 22.26 kg/m^3^ (n = 28, 35.40%), and the 95th percentile was 26.00 kg/m^3^ (n = 30, 38.00%). For male participants, the mean TMI was 18.10 ± 4.00 kg/m^3^, with the 85th percentile for TMI being 23.1 kg/m^3^ (n = 12, 33.30%), and the 95th percentile being 25.60 kg/m^3^ (n = 13, 36.10%).

The biomarkers of metabolic health are reported in Table 2. The mean HbA1c was 9.10% ± 2.80. The lipid profile demonstrated a total cholesterol of 4.30 ± 0.90 mmol/L, triglycerides 1.80 ± 1.90 mmol/L, LDL 2.30 ± 0.60 mmol/L, HDL 1.20 ± 0.40 mmol/L and ApoB:ApoA ratio 0.70 ± 0.20. Liver enzymes were assessed for detection of non-alcoholic fatty liver disease (NAFLD) including ALT (29.30 ± 27.60 U/L), AST (22.90 ± 17.50 U/L) and GGT (25.50 ± 17.40 U/L).

**Table 2:** Biomarkers of metabolic health in study participants.

Variable	Total (mean, SD)	Female (mean, SD)	Male (mean, SD)
HbA1c (%)	9.10 (2.80) (n = 116)	9.00 (2.60) (n = 80)	9.30 (3.10) (n = 36)
Total cholesterol (mmol/L)	4.30 (0.90) (n = 114)	4.20 (0.90) (n = 79)	4.40 (1.00) (n = 35)
Triglycerides (mmol/L)	1.80 (1.90) (n = 114)	1.80 (2.10) (n = 79)	1.80 (1.30) (n = 35)
LDL (mmol/L)	2.30 (0.60) (n = 111)	2.20 (0.60) (n = 77)	2.40 (0.70) (n = 34)
HDL (mmol/L)	1.20 (0.40) (n = 114)	1.20 (0.40) (n = 79)	1.20 (0.20) (n = 35)
ApoB:ApoA ratio	0.70 (0.20) (n = 60)	0.60 (0.20) (n = 43)	0.70 (0.10) (n = 17)
AST (U/L)	23.00 (18.00) (n = 107)	23.00 (14.00) (n = 74)	24.00 (24.00) (n = 33)
ALT (U/L)	29.00 (28.0) (n = 110)	28.00 (23.00) (n = 76)	33.00 (37.00) (n = 34)
GGT (U/L)	26.00 (17.00) (n = 109)	23.00 (17.00) (n = 77)	31.00 (17.00) (n = 32)
CRP (mg/L)	4.40 (3.70) (n = 100)	4.50 (3.60) (n = 70)	4.30 (4.00) (n = 30)

*HbA1c* hemoglobin A1c, *LDL* low-density lipoprotein, *HDL* high-density lipoprotein, *ApoB* apolipoprotein B, *ApoA* apolipoprotein A, *AST* aspartate transaminase, *ALT* alanine transaminase, *GGT* gamma-glutamyl transferase, *CRP* C-reactive protein, *SD* standard deviation.

All participants received lifestyle modification intervention. The majority of participants were on insulin therapy (n = 103), while 10 were on metformin, two on glyburide, and one on gliclazide. The treatment choice reflected the glycemic control status.

### The association of TMI and BMI z-score with DXA-measured adiposity

**Figure 1:**
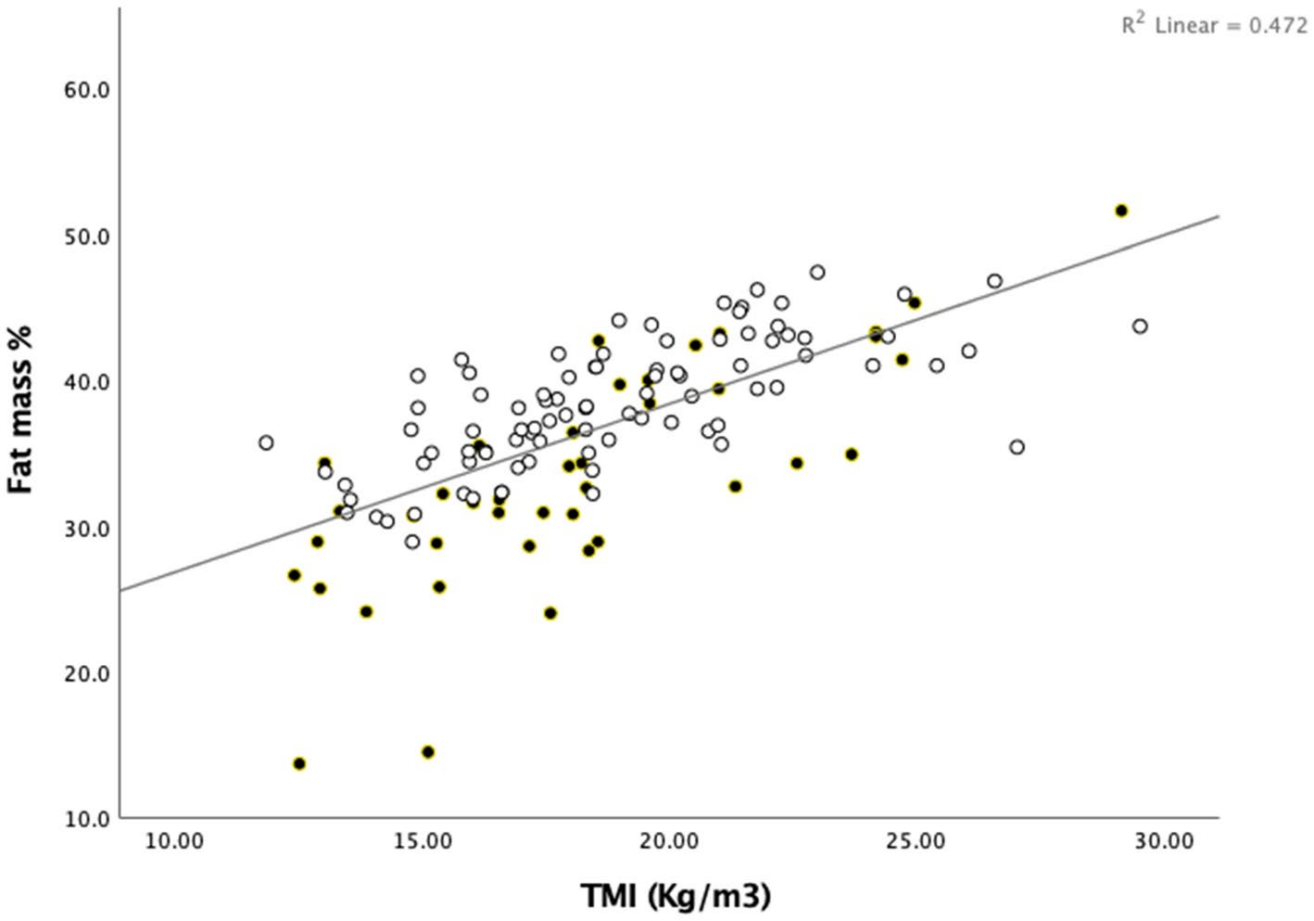
The relationship between DXA-based fat mass percentage and the TMI. *DXA* dual-energy X-ray absorptiometry, *TMI* tri-ponderal mass index. Clear circles=female, filled circles=male

To assess the strength of the relationship between the clinical and DXA-based measures of adiposity, we performed correlation analyses (Table 3, Fig. 1). TMI correlated strongly with total adiposity (FM%; r = 0.74, p-value < 0.001). In addition, TMI correlated strongly with WHtR (r = 0.85, p-value < 0.001) and to a lesser degree with WHR (r = 0.26, p = 0.005).

**Table 3:** Spearman’s correlation of TMI with BMI z-scores, measures of adiposity, lipid profile, and MAP dipping.

Variable	BMI z-score p-value (n)	TMI p-value (n)	FM % p-value (n)	WHR p-value (n)	WHtR p-value (n)
BMI z-score	–	0.09 p = 0.378 (n = 111)	− 0.08 p = 0.403 (n = 110)	0.19 p = 0.050 (n = 110)	0.11 p = 0.267 (n = 110)
TMI	0.09 p = 0.378 (n = 111)	–	0.74 p < 0.0001 (n = 115)	0.26 p = 0.005 (n = 115)	0.85 p < 0.0001 (n = 115)
FM %	− 0.08 p = 0.403 (n = 110)	0.74 p < 0.0001 (n = 115)	–	0.12 p = 0.198 (n = 114)	0.71 p < 0.0001 (n = 114)
WHR	0.19 p = 0.050 (n = 110)	0.26 p = 0.005 (n = 115)	0.12 p = 0.198 (n = 114)	–	0.43 p < 0.0001 (n = 115)
WHtR	0.11 p = 0.267 (n = 110)	0.85 p < 0.0001 (n = 115)	0.71 p < 0.0001 (n = 114)	0.43 p < 0.0001 (n = 115)	–
Total cholesterol	0.02 p = 0.881 (n = 109)	− 0.10 p = 0.269 (n = 114)	− 0.12 p = 0.196 (n = 113)	0.03 p = 0.751 (n = 113)	− 0.08 p = 0.398 (n = 113)
Triglycerides	− 0.08 p = 0.414 (n = 109)	0.06 p = 0.552 (n = 114)	0.06 p = 0.557 (n = 113)	0.17 p = 0.081 (n = 113)	0.08 p = 0.404 (n = 113)
LDL	0.08 p = 0.431 (n = 107)	0.04 p = 0.691 (n = 111)	−0.001 p = 0.992 (n = 110)	0.04 p = 0.715 (n = 110)	0.05 p = 0.580 (n = 110)
HDL	− 0.05 p = 0.621 (n = 109)	− 0.26 p = 0.005 (n = 114)	− 0.21 p = 0.024 (n = 113)	− 0.20 p = 0.031 (n = 113)	− 0.23 p = 0.017 (n = 113)
ApoB:ApoA	0.18 p = 0.181 (n = 55)	0.03 p = 0.833 (n = 60)	− 0.04 p = 0.768 (n = 59)	0.20 p = 0.122 (n = 59)	0.04 p = 0.741 (n = 59)
MAP dipping	− 0.07 p = 0.467 (n = 102)	− 0.10 p = 0.305 (n = 107)	− 0.21 p = 0.030 (n = 106)	0.02 p = 0.834 (n = 106)	− 0.26 p = 0.007 (n = 106)

*BMI* body mass index, *TMI* tri-ponderal mass index, *FM%* fat mass percentage, *WHR* waist-to-hip ratio, *WHtR* waist-to-height ratio, *LDL* low-density lipoprotein, *HDL* high-density lipoprotein, *ApoB* apolipoprotein B, *ApoA* apolipoprotein A, *HbA1c* hemoglobin A1c, *MAP* mean arterial pressure.

**Figure 2:**
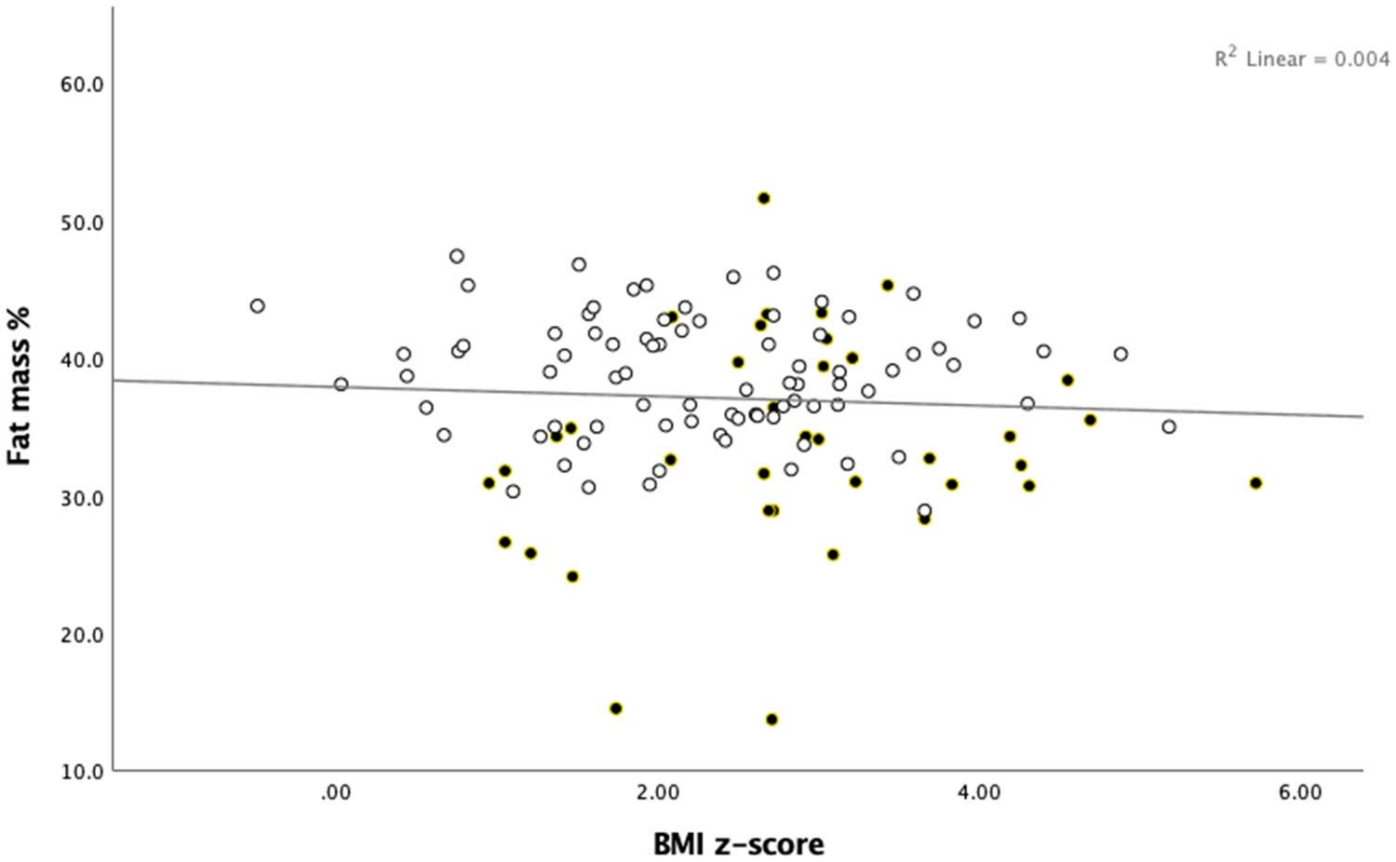
The relationship between DXA-based fat mass percentage and BMI z-score. *DXA* dual-energy X-ray absorptiometry, *BMI* body mass index. Clear circles=female, filled circles=male

The BMI z-score did not significantly correlate with the FM% (r = − 0.08, p-value 0.403) (Fig. 2), and had a weak but positive correlation with WHR (r = 0.19, p-value = 0.050). The BMI z-score did not correlate with the WHtR (r = 0.11, p-value = 0.267). Of note, TMI did not correlate with BMI z-score (r = 0.09, p-value 0.378). In summary, the TMI demonstrated a stronger correlation with total and central adiposity measures than BMI z-score.

### The association of TMI with components of the metabolic syndrome

To assess the association of TMI with components of the metabolic syndrome, we performed correlation analyses. The TMI had a small negative correlation with HDL levels (r = − 0.26, p-value = 0.005), but did not correlate with other components of the lipid profile including total cholesterol, LDL, triglycerides, and ApoB:Apo A (Table 3).

The multivariable regression analysis conducted to determine the association of TMI with central adiposity, hypertension, and dyslipidemia revealed that TMI was associated with WHtR (B = 35.54, 95% CI 28.81, 42.27, p-value < 0.0001), MAP dipping (B = 1.73, 95% CI 0.12, 3.33, p-value = 0.035), and HDL (B = − 5.83, 95% CI − 10.13, − 1.54, p-value = 0.008) (Table 4).

**Table 4:** Multivariable regression analysis of TMI adjusted for age, sex, MAP dipping, HDL and WHtR.

Variable	Unstandardized coefficients (B)	95% CI	p-value
Age (years)	− 0.17	− 0.41, 0.07	0.160
Sex	0.43	− 0.62, 1.47	0.420
MAP dipping	1.73	0.12, 3.33	0.035
WHtR	35.54	28.81, 42.30	< 0.0001
HDL	− 5.83	− 10.13, − 1.54	0.008

*MAP* mean arterial pressure, *HDL* high-density-lipoprotein, *WHtR* waist-to-height ratio.

While the FM% and WHtR were inversely correlated with mean arterial pressure (MAP) dipping (FM% r = − 0.21, p-value 0.030; WHtR r = − 0.26, p-value 0.007; Table 3), the regression analysis adjusting for age, sex, and HDL demonstrated no significant associations of these adiposity measures and MAP dipping (FM% B = − 0.69, 95% CI − 3.57, 2.19, p-value = 0.636; WHtR B = − 0.03, 95% CI − 0.09, 0.02, p-value = 0.205).

Taken together, these data demonstrate that TMI has stronger association with measures of total and central adiposity when compared to BMI z-score. In addition, TMI correlated with components of the metabolic syndrome frequently noted in T2DM patients.

## Discussion

The emergence of T2DM in youth is a global phenomenon that has been accelerating over the past few years. As diabetes is a major driver of adverse cardiometabolic outcomes in the general population, and youth with T2DM are expected to live longer with their diagnosis than their adult counterparts, there is an urgent need to define the drivers of cardiometabolic outcomes in this population. While adiposity is a major driver of cardiometabolic risk in the general population, its measurement has relied on using technologies that are not widely accessible. Compared to DXA scan-based adiposity measurement, TMI has emerged as a novel clinical tool to measure adiposity in adolescents with T2DM.

TMI has already been validated against DXA and bioelectrical impedance as an adiposity measurement tool in healthy pediatric populations, and our results corroborate its value as an adiposity measure in youth with T2DM^27–29^.

The use of TMI to measure adiposity in pediatric populations offers several advantages. While the BMI z-score is the most widely used surrogate marker of adiposity in children, this approach was adopted from BMI use as a marker of adiposity in adult populations. Adult BMI relies on the regression of weight on a constant height squared, which has some caveats yet is the most widely used measure to define obesity in population studies and clinical care settings^30^. However, the three dimensional nature of growth in children, including height gain, makes the regression of weight to height cubed, as with TMI calculation, a more accurate measure of adiposity^31^. In addition, TMI cut-offs are age-independent and sex-specific. Having a constant value in children and adolescents, although its value can be population-specific to define normal and excess adiposity, is an important criterion to assess adiposity during childhood^27,29,32^.

One of the important benefits of using TMI as a measure of adiposity is that its components are generated using stadiometers and weight scales, devices that are already part of routine pediatric clinical practice. This ease of calculation provides a powerful practical measure of adiposity that is based on routine anthropometric testing in the clinical setting and helps avoid the need for specialized and costly methodologies. In addition, the estimation of adiposity using TMI improves at higher fat mass levels^27^, which makes it an effective measure in the T2DM population as they typically have significant adiposity^33^.

TMI correlated with WHR and WHtR, important central adiposity measures, and with other metabolic syndrome components including blood pressure and HDL. The WHtR is one of the most reliable clinical measures of central adiposity^34,35^, which is associated with cardiometabolic disorders^12,15,17,18,36^.

The association of TMI with measures of central adiposity is an important finding, as previous evidence linked TMI to total adiposity assessments only. This may potentially allow the use of TMI as a prediction tool for metabolic syndrome and cardiometabolic comorbidities in T2DM patients^37,38^; however, this requires further validation.

A limitation of this study is that the use of WHtR ratio, a surrogate marker of central adiposity, was not validated against more accurate methods such as magnetic resonance imaging (MRI). The cost of such modalities is an important consideration in determining the feasibility of their measurement, yet it would be an important question to address in future studies.

TMI cut offs for determining overweight and obesity are calculated from the specific population under study. In our sample that is primarily composed of Indigenous youth in Canada, the generalizability of the cut offs proposed in our study may not be applicable to other populations, and further studies are required to determine the appropriate cut off for overweight and obesity for different ethnic groups. In addition, the cross-sectional nature of the study limits the determination of TMI as a tool to predict future cardiometabolic outcomes in this population.

Longitudinal data from a healthy pediatric population assessed whether childhood TMI can predict adult cardiometabolic risk. The TMI was associated with adult obesity, T2DM, high low-density lipoprotein, and increased carotid intima-media thickness^39^. However, the TMI performed equally well to BMI. It is uncertain whether the use of TMI in a population that already has T2DM during childhood may have higher predictive ability of future cardiometabolic diseases. This will require longitudinal follow-up data for the children with T2DM.

In conclusion, TMI is associated with total and central adiposity as well as markers of the metabolic syndrome in pediatric T2DM. TMI facilitates the measurement of adiposity in the clinical setting and, with further validation, may also be a useful longitudinal measure of future cardiometabolic risk prediction in pediatric T2DM patients.

## Methods

This is a cross-sectional secondary data analysis from the Improving Renal Complications in Adolescents with Type 2 Diabetes Through REsearch (iCARE) cohort Study, a national study that is assessing renal outcomes in children with T2DM in Canada. The data in this analysis are limited to the original site in Manitoba. The published study protocol reports further details regarding study procedures^40^.

The study has been approved by the Health Research Ethics Board, University of Manitoba and follows the relevant national and international regulations of human research studies. All participants and/or their guardians provided written informed consent and assent.

### Study participants

Patients with T2DM aged 10.20–17.90 years were included in this secondary data analysis (n = 116). The participants were recruited from the diabetes and nephrology clinics in Winnipeg, Manitoba, Canada.

The diagnosis of diabetes was based on the Diabetes Canada diagnostic criteria and supported by clinical criteria and the absence of diabetes associated autoantibodies^41^.

The study excluded patients with medication- or surgery-related diabetes, genetic forms of diabetes, and the presence of type 1 diabetes-related autoantibodies. In addition, those with a diagnosis of cancer and alcohol or drug abuse were excluded, as well as cases where either the patient or their caregiver were unable or unwilling to provide voluntary informed assent/consent, respectively.

### Data collection

Demographic data collected included age at study visit, age at diagnosis, sex, and duration of diabetes. Anthropometric data collected included height, weight, waist circumference, and hip circumference. Height was measured using ‘Health o meter Professional” model # 500LK. Waist-to hip ratio and waist-to-height ratio were calculated from the primary data. Dual‐energy x‐ray absorptiometry (DXA) scans (Hologic, Bedford, MA) were performed to quantify percent body fat, total fat mass, trunk fat mass, and fat‐free mass.

Blood pressure was assessed using 24-h ambulatory blood pressure monitors (SpaceLabs, Washington, USA). The mean arterial pressure (MAP) dipping was the parameter chosen from the ambulatory blood pressure profile for our analysis. The loss of the physiologic drop in blood pressure during sleep, or non-dipping, is associated with increased cardiovascular risk^42^, increased urinary albumin excretion which is a surrogate marker of microvascular disease^43^, and increased arterial wall stiffness^44^. Additionally, individuals with a higher BMI are more likely to have a smaller decrease in overnight blood pressure readings^45^.

Glycated hemoglobin A1c (HbA1c) levels were analyzed on a Roche Cobas Integra 800 CTS at local Diagnostic Services Manitoba (DSM) laboratory (assay referenced to the Diabetes Control and Complications Trial standard), and poor glycemic control was defined as an HbA1c level > 9.00%^40^.

Additionally, total cholesterol, low-density lipoprotein (LDL), high-density lipoprotein (HDL), triglycerides (TG), apolipoprotein A (ApoA) and apolipoprotein B (ApoB) were measured to evaluate for dyslipidemia. Alanine aminotransferase (ALT), aspartate transaminase (AST) and gamma-glutamyl transferase (GGT) were measured to determine liver health and the potential presence of fatty liver disease. Samples were collected in the fasting state if possible; 20% of samples were collected in a non-fasting state.

### Statistical analysis

Continuous variables are reported as means (standard deviation), and categorical variables are reported as numbers (percentage). Data were tested for normality of distribution using the Shapiro–Wilk test, and the data were log-transformed if not normally distributed, which included BMI percentile, fat mass percentage, waist-to-hip ratio, MAP dipping, triglycerides, and HDL. Spearman’s correlation test was used to determine the relationship between the different variables including TMI, BMI z-score, fat mass percentage, waist-to-hip ratio, waist-to-height ratio, MAP dipping, and lipids.

Multivariable linear regression analysis was performed to examine the association between TMI with MAP dipping, WHtR, and HDL, with age and sex added to the model. TMI was set as the dependent variable, with age, sex, MAP dipping, WHtR, and HDL as independent variables. We excluded LDL and total cholesterol due to the collinearity with HDL detected using the variance inflation factor analysis, and this analysis was also applied to assess the collinearity between WHR and WHtR. Unstandardized coefficient (B) and their p-values were reported. Statistical significance was set at alpha of 0.05.

## Data Availability

The data used for statistical analysis are available from the corresponding author upon reasonable justification.
